# A new type II CHH neuropeptide involves ovarian development in the peppermint shrimp, *Lysmata vittata*

**DOI:** 10.1371/journal.pone.0305127

**Published:** 2024-08-01

**Authors:** Huiling Lan, Fang Liu, Li Lu, An Liu, Haihui Ye

**Affiliations:** State Key Laboratory of Mariculture Breeding, Fisheries College of Jimei University, Xiamen, China; Zhejiang University College of Life Sciences, CHINA

## Abstract

Type II crustacean hyperglycemic hormone (CHH) neuropeptides play diverse roles in crustaceans. In the hermaphrodite shrimp *Lysmata vittata*, two transcripts of type II CHHs (molt-inhibiting hormone/gonad-inhibiting hormone, MIH/GIH1 and MIH/GIH2) were identified by transcriptome sequencing, and MIH/GIH1 was later named *Lvit*-GIH1 for its inhibitory effect on ovarian development. Based on the high similarity of MIH/GIH2 to *Lvit*-GIH1, we named tentatively MIH/GIH2 as *Lvit-*GIH2 and explored the role of *Lvit*-GIH2 in ovarian development. The open reading frame (ORF) of *Lvit*-*GIH2* was 333 bp in length, encoding a precursor consisted of a 32-aa signal peptide and a 78-aa mature peptide, which shared high sequence similarity with the type II subfamily peptides in crustaceans. Notably, *Lvit*-*GIH2* was widely expressed in multiple tissues. The qRT-PCR findings indicated a rising trend in the expression of *Lvit*-*GIH2* from the male phase to the euhermaphrodite phase. Both RNA interference and addition of GIH2 recombinant proteins (rGIH2) experiments showed that *Lvit*-GIH2 suppressed *Lvit*-*Vg* expression in hepatopancreas and *Lvit*-*VgR* expression in ovary. To further investigate the role of *Lvit*-GIH2 in ovarian development, the RNA-sequence analysis was performed to examine the changes in ovary after addition of rGIH2. The results showed that the pathways (Cysteine and methionine metabolism, Apoptosis-multiple species, etc.) and the genes (*17bHSD8*, *IGFR*, *CHH*, etc.) related to ovarian development were negatively regulated by rGIH2. In brief, *Lvit*-GIH2 might inhibit the ovarian development in *L*. *vittata*.

## 1 Introduction

Analogous to the hypothalamic-pituitary system of vertebrates, the X organ-sinus gland complex (XO-SG) is an important neuroendocrine regulatory center in decapod crustaceans, which is known to produce many neuropeptides that control the growth, metabolism, gonadal development and other physiological processes [1]. The crustacean hyperglycemic hormone (CHH) superfamily is a group of XO-SG neuropeptides that regulate various physiological processes in crustaceans, including molting, regulation of blood glucose homeostasis, osmotic pressure and reproduction [2,3]. The members of CHH superfamily include crustacean hyperglycemic hormone (CHH), ion transport peptide (ITP), molt-inhibiting hormone (MIH), gonad/vitellogenesis-inhibiting hormone (GIH/VIH), and mandibular organ inhibiting hormone (MOIH) [4]. Based on the differences in gene and precursor peptide structures, the CHH family is further divided into type I (CHH, ITP) subfamily and type II (GIH/VIH, MIH, MOIH) subfamily [4]. The amino acid structure of type I CHH is characterized by the presence of a CHH precursor related peptide (CPRP) between the signal and mature peptides, and the genes are composed of 4 exons and 3 introns [4]. Type I CHH mainly promotes carbohydrate utilization in hepatopancreas via stimulating glycolysis and lipolysis [5]. Besides, some reports also indicate that CHH inhibits testicular development by negatively regulating the insulin-like androgenic gland hormone (IAG) [6,7]. ITP is considered to be a crucial endocrine regulator of thirst and excretion with inhibition of feeding, regulation of thirst and excretion and transit of the meal throughout the digestive tract in insecta [8,9]. Unlike the Type I CHH, the genes of type II CHH are composed of 3 exons and 2 introns, which lacks of CPRP [4]. Among the type-II peptides, MIH inhibits the molting process by inhibiting the synthesis of ecdysone in the Y organ [10]. MOIH regulates reproduction, molting and metamorphosis by suppressing the secretion of methyl farnesate (MF) in the mandibular organ [11].

GIH/VIH also belongs to the type II CHH subfamily, which is considered to be the most effective inhibitor of vitellogenesis. It suppresses the ovarian development by inhibiting the synthesis of vitellogenin (Vg) in ovary and hepatopancreas [12]. In the Pacific white shrimp *Penaeus vannamei*, knockdown of *GIH* remarkedly increased *Vg* expression in hepatopancreas [13]. In the black tiger prawn *Penaeus monodon* and *P*. *vannamei*, knockdown of *VIH* could also significantly promote *Vg* expression in ovary and oocyte growth [14,15]. Besides, administration of VIH recombinant protein reduced the expression level of *Vg* mRNA in the cultured ovarian tissue of the American lobster *Homarus americanus* [16]. In the oriental river prawn *Macrobrachium nipponense*, RNA interference of *GIH* significantly promoted ovarian maturation [17]. Furthermore, GIH was also suggested to negatively regulate the expression of *IAG*, which plays a vital role in male sexual differentiation, metabolism, and growth in *M*. *nipponense* [18,19].

The peppermint shrimp, *Lysmata vittata* is a typical hermaphrodite crustacean species. Based on the previous research, the sexual development pattern of *L*. *vittata* is characterized by the unique protandric simultaneous hermaphroditism (PSH) sexual system whereby the gonad (ovotestis) is divided into ovarian region (anterior region) and testicular region (posterior) in dorsal view [20]. Two developmental phases (male phase and euhermaphrodite phase) involving four gonad developmental stages (stage I, II, III and IV) are defined for *L*. *vittata* based on the morphological and histological features [20]. Shrimp are functional male during the male phase (stage I, II, and III), whereby the testicular region of the gonad becomes sequentially mature whereas the ovarian region is still immature. Then they perform bigender function with the maturation of ovarian region during the euhermaphrodite phase (stage IV). Specifically, both regions are transparent at stage I, and become cloudy white at stage II. Shrimp show rapid ovarian development at stage III, whereby ovarian region changes from cloudy white to earthy brown with the accumulation of large amounts of vitellogenin. Testicular region is still cloudy white and filled with mature germ cells. At stage IV, ovarian region is fully developed and turns green. Two transcripts of type II CHHs (MIH/GIH1 and MIH/GIH2) in *L*. *vittata* were identified in the previous studies [21,22]. MIH/GIH1 was renamed as *Lvit*-GIH1 for its suppression on ovarian development by inhibiting *Lvit-Vg* expression in hepatopancreas [22]. As the role of MIH/GIH2 has not yet been clarified, it is still unclear whether MIH/GIH2 is also a functional GIH in *L*. *vittata*.

In the present study, preliminary investigation showed that MIH/GIH2 demonstrated a high level of similarity to *Lvit*-GIH1. Moreover, the expression level of *MIH/GIH2* in the eyestalk ganglion showed an increasing trend from male phase to euhermaphrodite phase. Thus, we proposed that MIH/GIH2 might also possess GIH biological functions and designated tentatively it as *Lvit-*GIH2. In order to test the above conjecture, we explored the regulatory roles of *Lvit-*GIH2 in gonadal development via *in vitro* knockdown and overexpression experiments. The RNA-sequencing (RNA-seq) analysis was further performed to examine the changes in ovary after addition of GIH2 recombinant protein (rGIH2). The present study provided basic data for understanding the molecular mechanism of *Lvit-*GIH2 on ovarian development in *L*. *vittata*.

## 2 Materials and methods

### 2.1 Animals

The shrimp (*L*. *vittata*) used in this study were collected from Taizhou coastal area, Zhejiang Province, China, and then artificial-bred at the Fisheries College of Jimei University in Xiamen city. The seawater temperature was 26 ± 0.5°C and the salinity was 30 ± 2 PSU. The shrimp were fed twice daily with shrimp feeds regularly. Animal use was approved by the Animal Care and Use Committee of the Fisheries College of Jimei University.

### 2.2 Coding sequence cloning of *Lvit-GIH2*

Total RNA was extracted from eyestalk ganglia of *L*. *vittata* with Magzol reagent (Megan, China) according to the manufacturer’s instructions. First-strand of cDNA was synthesized by PrimeScript™ RT reagent Kit with gDNA Eraser (Perfect Real Time) (Takara, Japanese). *Lvit-*GIH2 transcript was obtained from the previous study [21]. Polymerase chain reaction (PCR) was the performed by specific primer pair (GIH2F/GIH2R) to clone the open reading frame (ORF) of *Lvit-*GIH2. The ORF was predicted by ORF Finder (https://www.ncbi.nlm.nih.gov/orffinder/). Primer pairs GIH2-F/GIH2-R were designed by Primer Premier 5.0 software to verify the selected *Lvit-GIH2* sequence (Table 1).

**Table 1:** Summary of primers used in this study.

Primer	Primer sequence(5’-3’)	Application
GIH2-F	CAAGCATAAAAATGGTTTTCA	PCR
GIH2-R	AATGTAGTCCGCTCACTTC	PCR
GIH2-QF	GAAGTGAGCGGACTACATT	qRT-PCR
GIH2-QR	ATATGGCGTGTGGTTCTG	qRT-PCR
GIH2-PF	CGCGGATCCGCGAGATACCTAGACGACGAATG	qRT-PCR
GIH2-PR	CCCAAGCTTGGGCTTCCTGCCGGCCTTGAGAA	qRT-PCR
GIH2-dsF	ATGGTTTTCAGCACAACTCCA	qRT-PCR
GIH2-dsR	AAGATCTCGGTTTCCCATCAC	qRT-PCR
Vg-QF	CTGCGTCTCGGAACTCAA	qRT-PCR
Vg-QR	GTGCTGGTGGTGAAGATGA	qRT-PCR
VgR-QF	TCTTCTATTAACACACAGGTG	qRT-PCR
VgR-QR	CTCTGCCATCCAAACCACAT	qRT-PCR
GAPDH-QF	TGAAGTCGCAGGATACAACA	qRT-PCR
GAPDH-QR	TGAAGTCGCAGGATACAACA	qRT-PCR
CHH-F	AAGGACTGGCAAGGATT	qRT-PCR
CHH-R	GAAGAGTTCACGGTCATAGA	qRT-PCR
17bESD8-F	CGCTATCGTGGTGTCG	qRT-PCR
17bESD8-R	GGCTTGGGATGTCAGG	qRT-PCR
GPL-F	TGGTAAAGGACAGAAACG	qRT-PCR
GPL-R	CTGACTGGATGCCTAAAT	qRT-PCR
IGFR-F	CCCTGAAGCCCTCTGT	qRT-PCR
IGFR-R	GCCATAAGGACCAACTGT	qRT-PCR
COX-F	CTACTGACGCAGGAAACC	qRT-PCR
COX-R	CATGGCAAGGAATAAGC	qRT-PCR
Orct-F	TTCGTGAGGGATTATGAG	qRT-PCR
Orct-R	GATGATCGTGCGGTATT	qRT-PCR
PARGL-F	GAAGAGGAGGAACGAGAC	qRT-PCR
PARGL-R	AGCATTGGCAAGGAGA	qRT-PCR
Parp3-L-F	TCGGAGAATCAGGTCAG	qRT-PCR
Parp3-L-R	TCCCTTTTAAGGTTGGTG	qRT-PCR

### 2.3 Analysis of the nucleotide and deduced amino acid sequences

*Lvit-*GIH2 transcript was obtained from the previous study [21]. Signal peptides were predicted by SignalP 5.0 (https://services.healthtech.dtu.dk/services/SignalP-5.0/). Amidation sites were predicted by NetNGlyc 1.0 Server (https://services.healthtech.dtu.dk/services/NetNGlyc-1.0/). Multiplex sequence alignment of MIH/GIH sequences was carried out with known sequences of other species to generate multiplex sequence alignments of MIH/GIH sequences by Clustal Omega (https://www.ebi.ac.uk/jdispatcher/msa/clustalo). A phylogenetic tree was then constructed by MEGA6 software using Neighbor-Joining (NJ) method. Sequences used in multiplex sequence alignments and phylogenetic tree analysis were list in Table 2.

**Table 2:** Summary of sequences used in multiple sequence alignment and phylogenetic analysis.

Sequence	Species	GenBank accession number
Lvit-GIH2	Lysmata vittata	MT313290.1
Lvit-GIH1	Lysmata vittata	MT113121.1
*Pv-*GIH	Penaeus vannamei	AHJ11242.1
*Pem*-GIH	Penaeus monodon	ABG33898.1
*Pem*-VIH	Penaeus monodon	UIW23014.1
*Ha*-GIH	Homarus americanus	CAA60644.1
*So*-VIH	Scylla olivacea	AZF98733.1
*Cq*-MIH	Cherax quadricarinatus	ACX55057.1
*Mee*-MIH	Metapenaeus ensis	AAC27452.1
*Nn*-MIH	Nephrops norvegicus	QBX89053.1
*Pc*-MIH	Palaemon carinicauda	AVI44922.1
*Mn*-MIH	Macrobrachium nipponense	AJM70548.1
*Cm*-MIH	Carcinus maenas	CAA53591.1

### 2.4 Tissue distribution of *Lvit-GIH2* mRNA in *L*. *vittata*

The mRNA expression in various tissues (eyestalk ganglion, brain, thoracic ganglion, ventral ganglion, heart, ovary, testis, androgenic gland, hepatopancreas, stomach, intestine, gill and muscle) of *L*. *vittata* at stage IV gonadal development (n = 3) was detected by reverse transcription-PCR (RT-PCR) with primer pair (GIH2-QF/GIH2-QR). Total RNA extraction and the synthesis of cDNA were performed as described in Section 2.2. The PCR reaction was performed under the following conditions: 95°C for 3 min; 32 cycles of 95°C for 30 s, 58°C for 30 s and 72°C for 30 s; and an extension at 72°C for 5 min. *Lvit-β-actin* (GenBank accession number: MT114194) was amplified as a positive control. PCR products were finally analyzed by 1.5% agarose gel electrophoresis and imaged by Gel Image System (Tanon, China).

### 2.5 Expression profile of *Lvit-GIH2* during gonadal development

Total RNA was extracted from eyestalk ganglion of *L*. *vittata* at male phase and euhermaphrodite phase (n = 5), followed by synthesis of cDNA as described above (Section 2.2). The expression profile of *Lvit-GIH2* during gonadal development was investigated by qRT-PCR. Each cDNA template was amplified with primer pair (GIH2-QF/GIH2-QR), and *Lvit-GAPDH* (GenBank accession number: MK471093.1) was utilized as internal control. The qRT-PCR system contained 2 μL cDNA, 0.6 μL of each primer 6.8 μL H_2_O, PerfectStart® Green qPCR SuperMix (Transgen Biotech, China) 10 μL, and totaled 20 μL. The reaction was performed in QuantStudio5 (Applied Biosystems, America), and the PCR protocol was performed as follows: 95°C for 3 min; followed by 40 cycles of 95°C for 30 s, 58°C for 30 s and 72°C for 30 s. Results were calculated using 2^-ΔΔCt^ method.

### 2.6 *In vitro* experiment: Effect of *GIH2*-dsRNA administration on *Vg* expression in hepatopancreas and *VgR* expression in ovary

*Lvit-GIH2* fragment (144 bp) was cloned into pGEM-T Easy Vector (Promega, China). dsRNA synthesis was performed using T7 RNAi Transcription Kit (Vazyme, China) following the standard protocols. Shrimp at the stage IV of gonadal development were used for the experiment (n = 5). After anesthetization, the eyestalks, hepatopancreas and ovarian regions were dissected out of the shrimp. Hepatopancreas and ovarian regions were then evenly divided into 2 portions followed by being cleaned with the crustacean physiological saline containing penicillin (100 units/mL) and streptomycin (100 μg/mL) (Sangon, China). Tissues were then cultured in 24-well plates with 500 μL Leibovitz’s (1X) L-15 medium (Phenol Red free, GIBCO) at 26°C for 1 h. An ovarian tissue and an eyestalk were co-cultured in the same well, while a hepatopancreas tissue was cultured in a single well. After replacing the original medium by 500 μL medium containing *GIH2*-dsRNA at a concentration of 2 μg/g, the plates were incubated at 26°C for another 6 h. PBS was used as the negative control. The total RNA extraction, cDNA synthesis and qRT-PCR were performed as described in Section 2.2 and 2.5.

### 2.7 *In vitro* experiment: Effect of rGIH2 on *Lvit-Vg* expression in hepatopancreas and *Lvit-VgR* in ovary

The fragment encoding *Lvit*-GIH2 mature peptide was cloned into pET-His vector with restriction enzyme sites (BamH I and Hind III). The generated *Lvit*-GIH2-pET-His vector was introduced into the host BL21 (DE3) *E*. *coli* by transformation cultured in LB liquid medium containing 100 μg/mL ampicillin at 37°C for protein expression. When OD_600_ reached 1, isopropyl-β-D-thiogalactoside (IPTG) was added (1 mM final concentration) and cultured at 37°C for 8 h to induce protein expression. rGIH2 was purified with His-tag Protein Purification Kit (LABLEAD, China). Shrimp at the stage II of gonadal development were used for the experiment (n = 3). *In vitro* experiment was performed with reference to Section 2.5 containing rGIH2 at a concentration of 10^−8^ M. The total RNA extraction, cDNA synthesis and qRT-PCR were performed as described in Section 2.2 and 2.5.

### 2.8 cDNA library construction and transcriptome sequencing

In order to further explore the effects of *Lvit*-GIH2 on ovarian development, transcriptome sequencing was performed on ovarian explants applied with rGIH2 by Gene Denovo Biotechnology Co., Ltd (Guangzhou, China). The total RNA extraction was performed with reference to Section 2.2. Differential expression analysis of RNAs was performed by DESeq2 and edgeR. The genes with the parameter of false discovery rate (FDR) below 0.05 and absolute fold change ≥ 2 were considered differentially expressed genes (DEGs). The DEGs in ovaries were enriched by GO and KEGG analysis to explore the function. Hypergeometric distribution test was used to calculate the significance of differential gene enrichment in each pathway entry, and KEGG pathways with significant enrichment were selected.

Nine DEGs from significant signaling pathways in KEGG analysis were randomly selected to validate transcriptome sequencing outcomes. Primers were designed with PrimerPremier 5.0 software. The reliability of the results of RNA-seq was verified by qRT-PCR. The 2^-ΔΔCt^ method was used to analyze the target gene’s relative expression (fold changes). The synthesis of cDNA, qRT-PCR and statistical methods of data were conducted with reference to Section 2.2 and 2.5.

## 3 Results

### 3.1 Sequence cloning of *Lvit-GIH2*

**Fig 1:**
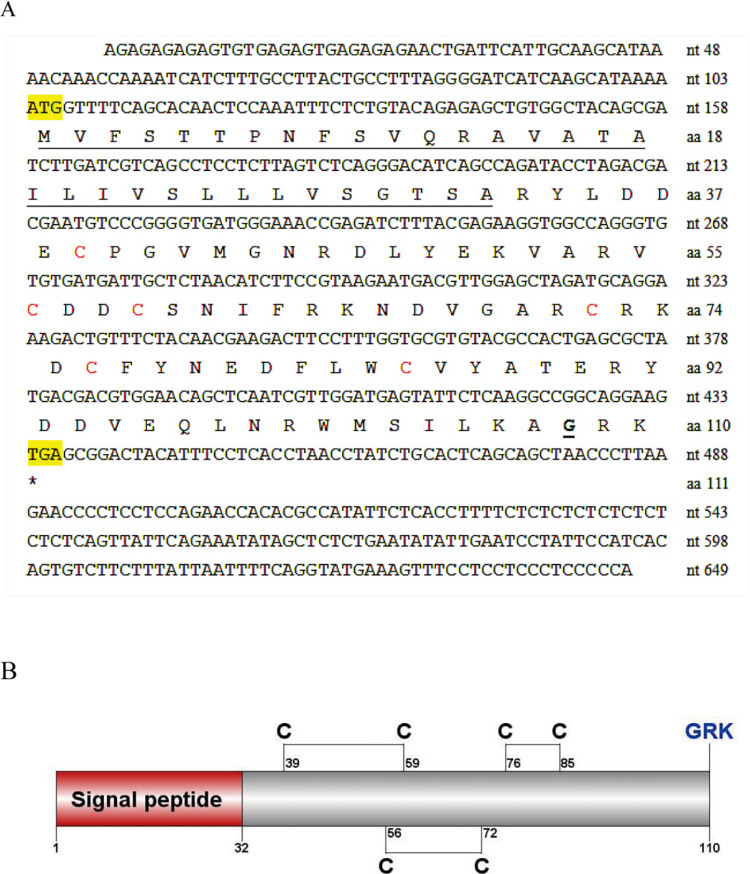
Molecular characterization of *Lvit-*GIH2. (A) The nucleotide and deduced amino acid sequence of *Lvit-*GIH2. The signal peptide is underlined, the initiation and termination codons are shown in yellow background, the six cysteine residues are shown in red and the assumed amidation site is underlined in bold format. (B) Schematic diagram of preproprotein of *Lvit-*GIH2. Signal peptide (red box), the mature hormone (gray box) and GRK (bold blue) are shown. The six cysteine residues are predicted, and three putative disulfide bridges are connected with lines.

The ORF of *Lvit-GIH2* (GenBank accession number: MT313290.1) was 333 bp in length, which encoded a precursor of a 32-aa signal peptide and a 78-aa mature peptide. Similar to the other members of type II CHH subfamily, six conserved cysteine residues were predicted at position 39, 56, 59, 72, 76 and 85 (Cys^39^, Cys^58^, Cys^59^, Cys^72^, Cys^76^, Cys^85^), forming three disulfide bridges. Also, a glycine was predicted at position 44 (Gly ^44^) (Fig 1).

### 3.2 Homology and phylogenetic analysis

Six conserved cysteine residues forming three disulfide bridges were observed in the mature peptide of type II CHHs. *Lvit-*GIH2 mature peptide shared the highest identity with MIH of *M*. *nipponense* (89.74%) and the ridgetail white prawn *Palaemon carinicauda* (89.74%), followed by *Lvit-*GIH1 of *L*. *vittata* (75.64%). Notably, *Lvit*-GIH1 also showed relatively higher amino acid sequence identity with the two MIH sequences mentioned previously (78.21% to *Mn*-MIH and 76.92% to *Pc-*MIH). Besides, *Lvit*-GIH1 has sequence similarity with *Lvit-*GIH2 by 75.64%, which is higher than most of sequences studied before (S1 Fig). The result of phylogenetic analysis revealed that there was no apparent clustering of these type II CHH sequences. *Lvit-*GIH2 was clustered into a single branch with the MIH of *P*. *carinicauda* and *M*. *nipponense*, but not with *Lvit-*GIH1, indicating that *Lvit-*GIH2 and *Lvit-*GIH1 belonged to the CHH type II subfamily but not the same neuropeptide (S2 Fig).

### 3.3 Tissue distribution and expression profile of *Lvit-GIH2* during gonadal development

**Fig 2:**
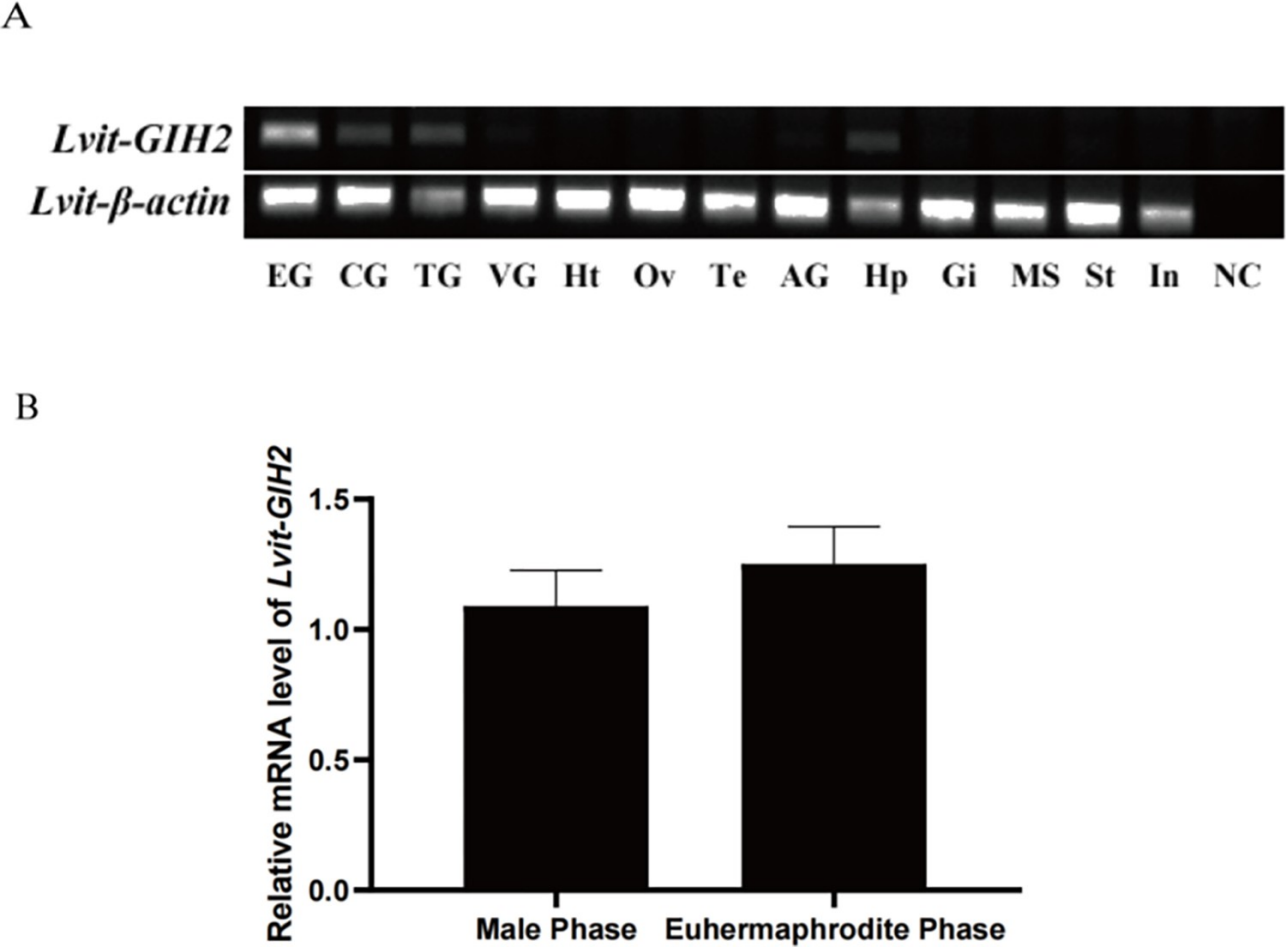
Spatial and temporal expression profiles of *Lvit-GIH2*. (A) Distribution of *Lvit-GIH2* in different tissues of *L*. *vittata*. The analysis is generated by RT-PCR assays with cDNAs from various tissues of individuals at the gonadal development stage IV (n = 3). The *Lvit-GAPDH* gene is used as a reference control gene. (B) Expression profile of *Lvit-GIH2* in the eyestalk ganglion during gonad development by qRT-PCR. The *Lvit-GIH2* expression levels standardized by *Lvit-GAPDH* expression levels are represented as mean± SEM (*t*-test; n = 5).

The RT-PCR results showed that *Lvit-GIH2* was widely expressed in the eyestalk ganglion, brain, thoracic ganglion, ventral ganglion, androgenic gland, hepatopancreas, stomach, intestine, gill and muscle. In addition, the qRT-PCR results showed that *Lvit-GIH2* was detected in the eyestalk ganglion with the increasing trend from male phase to euhermaphrodite phase (*P* > 0.05) (Fig 2).

### 3.4 *In vitro* experiment: Effect of *Lvit*-GIH2 knockdown

**Fig 3:**
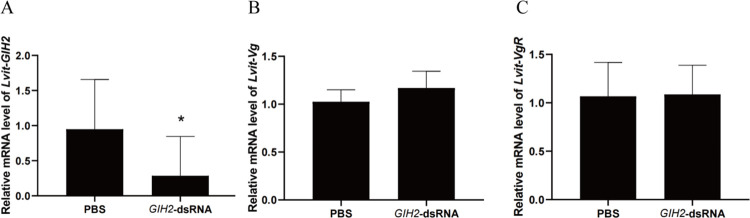
Effects of Lvit-GIH2 interference on gene expression of *L*. *vittata in vitro*. (A) *Lvit-GIH2* expression levels in the eyestalk (“*”, *P* < 0.05). (B) *Lvit-Vg* expression levels in hepatopancreas. (C) *Lvit-VgR* expression levels in the ovarian region. The gene expression levels standardized by *Lvit- GAPDH* expression levels are represented as mean ± SEM (*t*-test; n = 5).

The results showed that in comparison to the PBS group, the *Lvit-*GIH2 transcripts levels in eyestalk ganglion were significantly inhibited by 70% (*P* < 0.05) in GIH2-dsRNA group. The expression of *Lvit-Vg* and *Lvit-VgR* in the hepatopancreas and ovary, respectively, showed an upward trend by *GIH2*-dsRNA addition as compared to that of the PBS control (*P* > 0.05) (Fig 3).

### 3.5 *In vitro* experiment: Effect of rGIH2 administration

**Fig 4:**
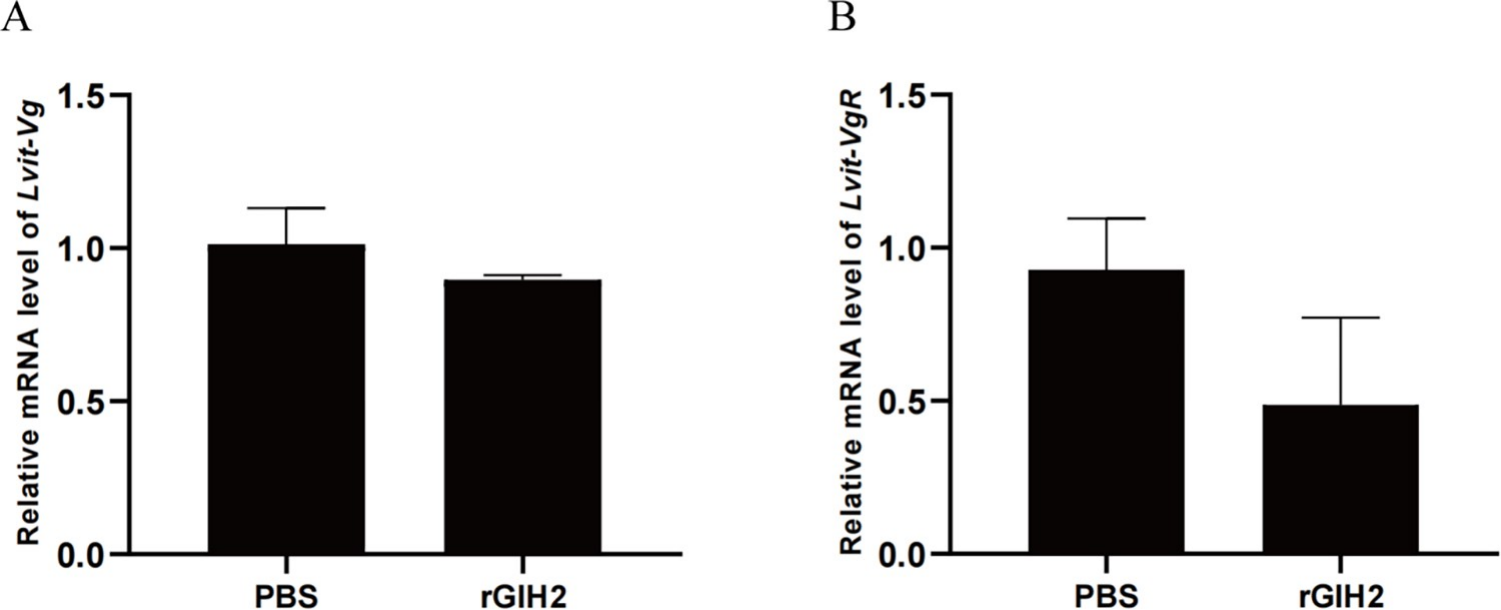
Effects of rGIH2 on gene expression of *L*. *vittata in vitro*. (A) *Lvit-Vg* expression levels. (B) *Lvit-VgR* expression levels. The gene expression levels standardized by *Lvit- GAPDH* expression levels are represented as mean ± SEM (*t*-test; n = 3).

The rGIH2 was obtained through a prokaryotic system (S3 Fig). Results showed that the abundance of *Lvit-Vg* in hepatopancreas (*P* > 0.05) and *Lvit-VgR* in ovary (*P* > 0.05) was slightly inhibited by rGIH2 at the concentration of 10^−8^ M as compared to that of the PBS control (Fig 4).

### 3.6 Transcriptome sequencing of ovary after rGIH2 administration

Transcriptome sequencing was further performed on ovary (OP VS OG) to elucidate the mechanism of GIH2 inhibiting ovarian development.

#### 3.6.1 Summary statistics of transcriptome sequencing and *de novo* assembly

After filtering out low quality reads using the Illumina HiSeq 3000, we obtained 36,187,904,341 bp clean reads for ovary. All samples were detected by FastP and Q20 values were higher than 98%, which indicated high quality of sequencing (Table 3). Clean reads were pooled and assembled into non-redundant transcripts without reference genomes using Trinity software.

**Table 3:** Transcriptome sequencing data of the ovary in *L*. *vittata*.

Sample	Raw reads	Clean reads	Q20(%)	Q30(%)	GC(%)
OP1	6,605,965,500	6,526,901,684	98.70%	96.08%	41.15%
OP2	5,560,455,300	5,500,783,687	98.64%	95.94%	41.22%
OP3	5,964,724,500	5,897,700,743	98.68%	95.98%	41.21%
OG1	5,716,331,100	5,663,067,247	98.70%	96.07%	41.74%
OG2	5,893,543,800	5,832,769,335	98.69%	96.10%	40.54%
OG3	6,840,025,500	6,766,681,645	98.78%	96.30%	40.57%

#### 3.6.2 Identification and functional analysis of DEGs

Compared with the PBS control, 1,517 DEGs including 1,051 up-regulated DEGs and 466 down-regulated DEGs were identified in ovary. 475 DEGs in ovary were aligned to the GO database. GO enrichment analysis classified gene clustering into three major functional categories: biological process, cellular component, and molecular function. Based on the overall annotation, the biological processes enriched the most entries (S4 Fig).

**Fig 5:**
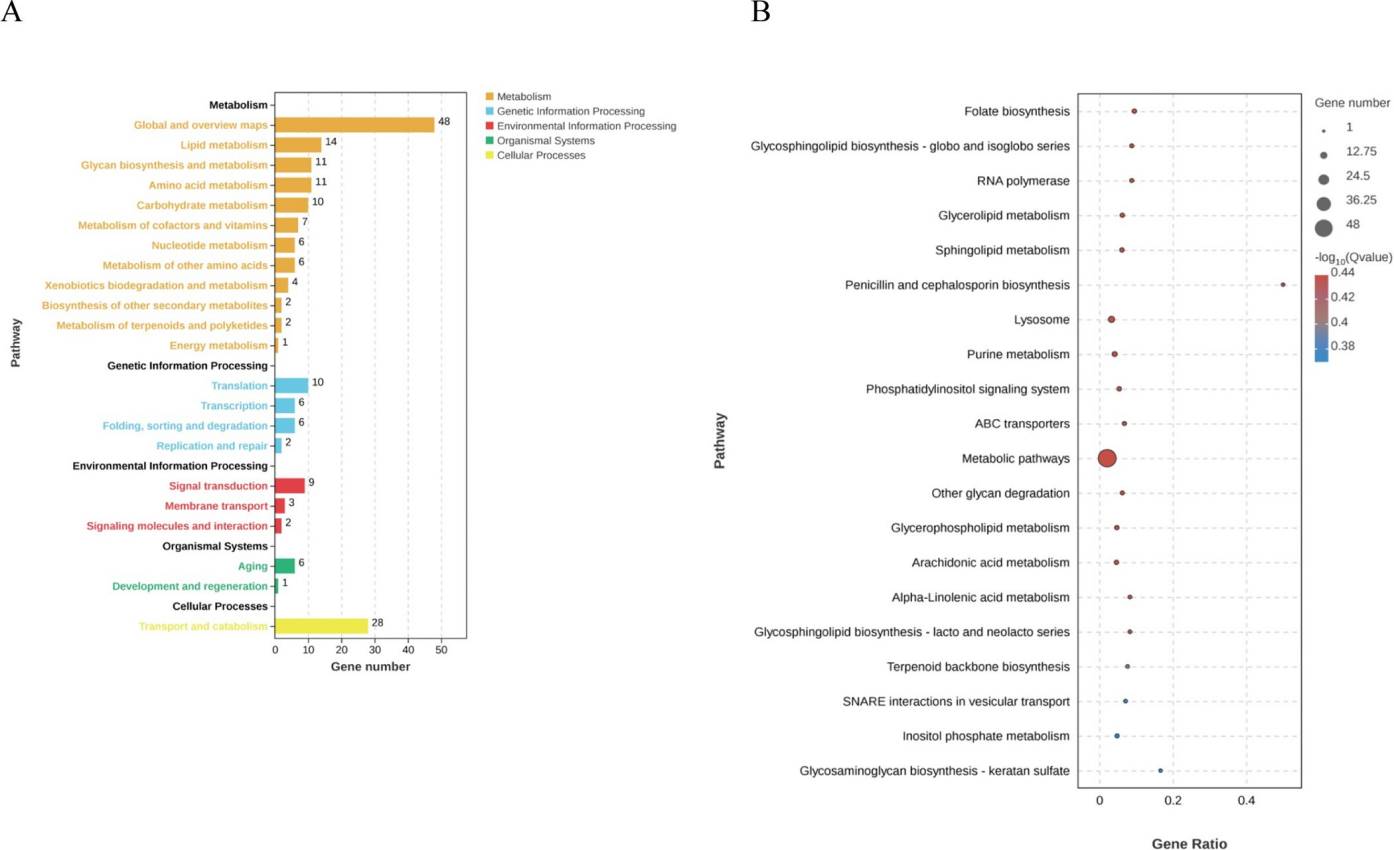
KEGG analysis of up-regulated DEGs. (A) KEGG pathways enriched by the DEGs. The x-axis indicates the significant level of enrichment, and the y-axis indicates the KEGG pathway. (B) Bubble map of top 20 the KEGG pathway enriched to DEGs sorted by *P*-value. The x-axis indicates the significant level of enrichment, and the y-axis indicates the KEGG pathway. The color gradient from blue to red corresponds to the enrichment significance from low to high.

**Fig 6:**
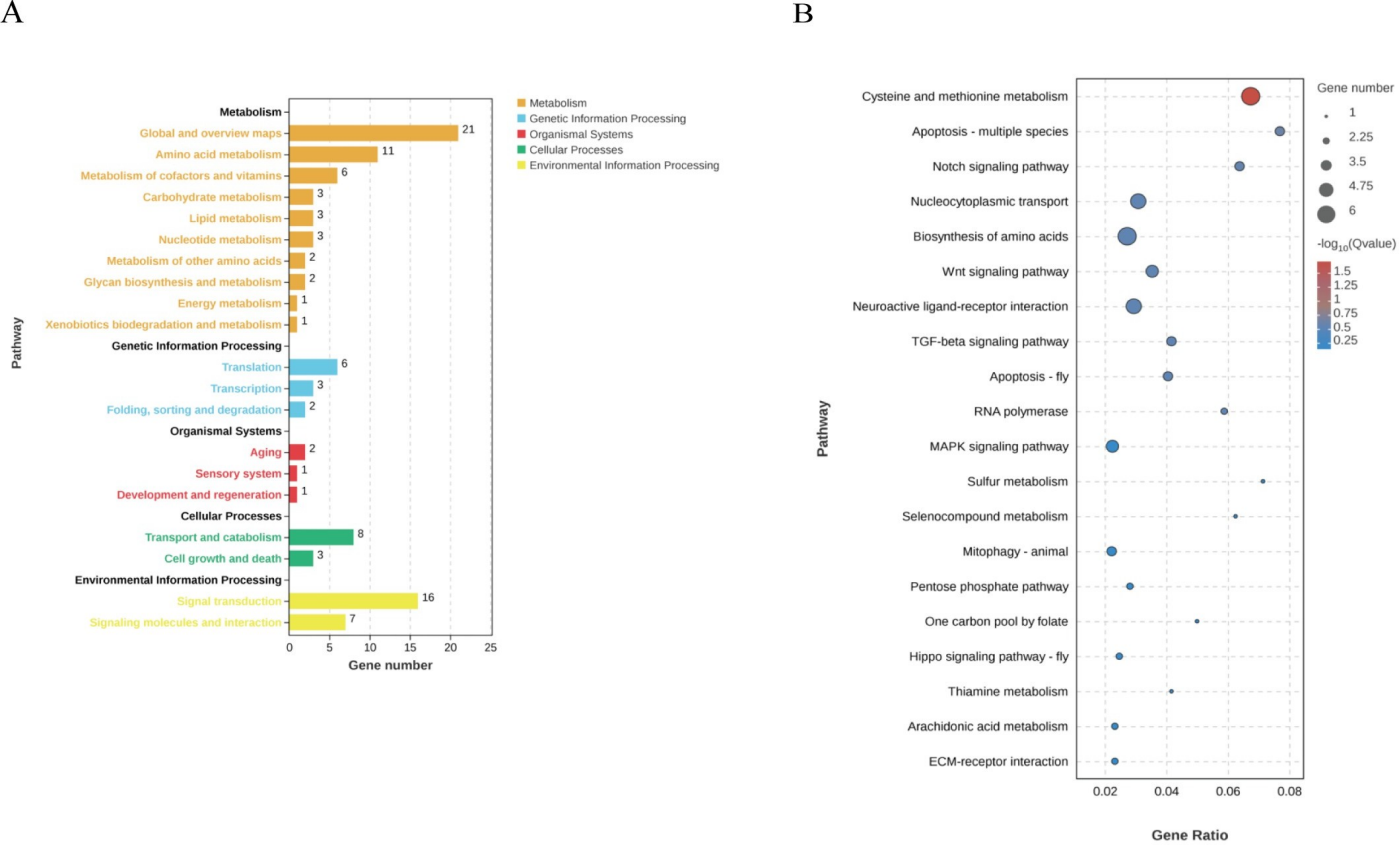
KEGG analysis of down-regulated DEGs. (A) KEGG pathways enriched by the DEGs. (B) Bubble map of top 20 the KEGG pathway enriched to DEGs sorted by *P*-value. The x-axis indicates the significant level of enrichment, and the y-axis indicates the KEGG pathway. The color gradient from blue to red corresponds to the enrichment significance from low to high.

KEGG analysis was applied to identify the biological pathways that are related to the unigenes. In total, 6,037 unigenes were highly matched to known genes and assigned to 108 signaling pathways. Up-regulated and down-regulated DEGs were subjected to KEGG analysis based on transcriptomic data, respectively. The results showed that the top 5 significant signaling pathways of up-regulated DEGs were Folate biosynthesis (42, 0.70%), Glycosphingolipid biosynthesis—globo and isoglobo series (34, 0.56%), RNA polymerase (34, 0.56%), Glycerolipid metabolism (64, 1.06%), and Sphingolipid metabolism (65, 1.08%) (Fig 5). The top 5 significant signaling pathways of down-regulated DEGs were Cysteine and methionine metabolism (89, 1.47%), Apoptosis-multiple species (39, 0.65%), Notch signaling pathway (47, 0.78%), Nucleocytoplasmic transport (162, 2.68%), and Biosynthesis of amino acids (220, 3.64%) (Fig 6).

**Fig 7:**
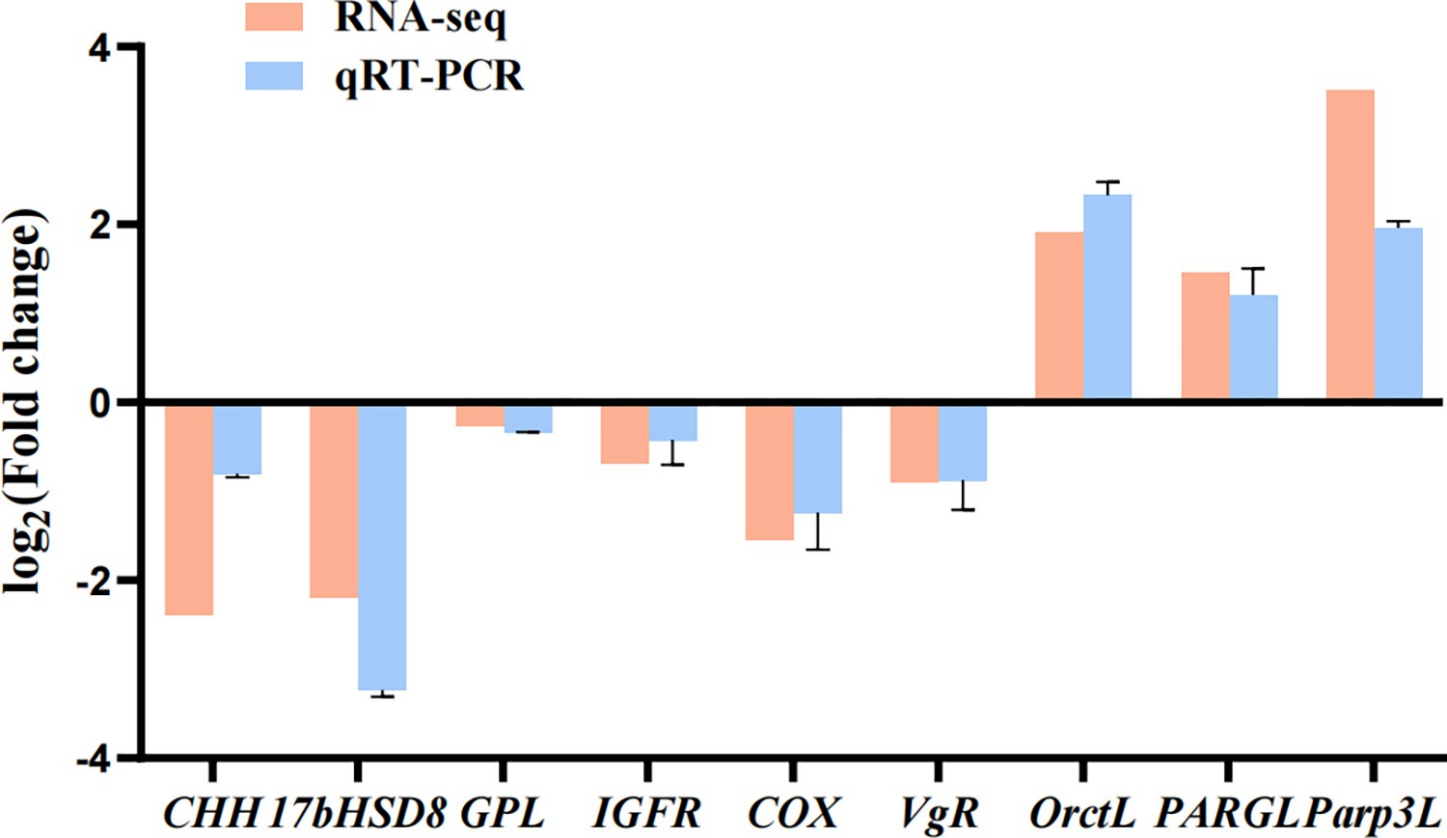
qRT-PCR validation of DEGs. Comparison of the expression profiles of selected genes as determined by RNA-seq and qRT-PCR.

**3.6.3 Screening and expression of genes related to ovarian development.** Nine DEGs from significant signaling pathways in KEGG analysis were randomly chosen for data verification of RNA-seq by qRT-PCR. The results showed that the expression levels of these genes were identical to the RNA-seq results, indicating that the transcriptomic data were reliable (Fig 7).

## 4 Discussion

It is known that type I CHH can be easily distinguished from type II CHH on the basis of differences in precursor peptides [4]. The precursor peptide of type I CHH consists of a signaling peptide, a preconditioning-related peptide (CPRP), and a maturation peptide, whereas the precursor peptide of type II CHH lacks the CPRP region [4]. The results of structure prediction showed that the precursor peptide of MIH/GIH2 only consisted of the signal peptide and the mature peptide, which suggested that structurally, MIH/GIH2 is categorized as type II CHH. But it is difficult to further distinguish GIH from MIH in the type II CHH family. In this study, results of multiple sequence alignment revealed that MIH/GIH2 had high sequence similarity with *Mn-*MIH, *Pc-*MIH, and *Pem-*VIH. Phylogenetic tree analysis also demonstrated that MIH/GIH2 was closely related to *Mn-*MIH and *Pc-*MIH. Interestingly, we found that *Lvit-*GIH1 exhibits a greater degree of similarity to MIH than to GIH, even though it has been confirmed to have inhibitory role in ovarian development in *L*. *vittata* [22]. Because MIH/GIH2 demonstrates a high level of similarity to *Lvit-*GIH1, we tentatively designated it as *Lvit-GIH2*.

Different from *Lvit-*GIH1 [22], *Mr-*GIH [23] and *Ha-*GIH [12], which are exclusively expressed in the eyestalk ganglion, *Lvit-GIH2* was widely expressed in several tissues as same as GIH in the sand lobster, *Panulirus homarus* [24]. In addition, the expression level of *Lvit-GIH2* in the eyestalk ganglion showed an increasing trend from male phase to euhermaphrodite phase in *L*. *vittata*, which is inconsistent with the results of several previous studies [12,23]. As GIH mainly suppresses the ovarian development by inhibiting the synthesis of vitellogenin, in its turn, *GIH* mRNA expression levels increase during ovarian development and decrease after ovarian maturation [12–16,24,25]. Similar expression trend was also observed for *Lvit-GIH1* in *L*. *vittata* [22]. Notably, in the oriental river prawn *M*. *nipponense*, RNA interference of *GIH* significantly promoted ovarian maturation, suggesting an inhibitory effect of GIH toward ovarian maturation [17]. It is assumed that *Lvit-*GIH2 might have other physiological roles besides vitellogenesis as *Lvit-*GIH1 did in the PSH species.

In order to test the above assumption, we carried out *in vitro* knockdown and overexpression experiments. Results demonstrated that following *Lvit-GIH2* knockdown, *Lvit-Vg* in hepatopancreas and *Lvit-VgR* in ovary showed a tendency of upregulation but not significantly. While in contrast, administration of rGIH2 *in vitro* resulted in downregulation of *Lvit-Vg* and *Lvit-VgR* expression. In *P*. *vannamei* and *P*. *monodon*, silencing of GIH significantly increased *Vg* mRNA expression in hepatopancreas [13–15]. After cultured with addition of VIH recombinant protein, expression of *Vg* mRNA in ovary decreased in *H*. *americanus* [16]. The above results confirmed that *Lvit-*GIH2 exercised the biological function of GIH and exerted an inhibitory effect on the expression of *Lvit-Vg* and *Lvit-VgR*.

In the oriental river prawn *M*. *nipponense*, GIH was also demonstrated to suppress ovarian maturation, indicating that GIH might regulate multiple physiological processes in ovaries [17]. We speculated that *Lvit-*GIH2 might have other physiological roles besides vitellogenesis. Thus, we performed the transcriptome sequencing on ovarian explants applied with rGIH2 to figure out its characters in ovarian physiology. Our results indicated that *Lvit-*GIH2 regulated several metabolic pathways in ovary. Results of DEG analysis indicated that *Lvit-*GIH2 was involved in several physiological processes. Down-regulated DEGs were much more than up-regulated DEGs in ovary. KEGG analysis demonstrated that the down-regulated DEGs mainly enriched in Cysteine and methionine metabolism, Nucleocytoplasmic transport, and Biosynthesis of amino acids. The Cysteine and methionine metabolism signaling pathway is a critical regulator of ovarian development, particularly in the down-regulation of DEGs enrichment. Cysteine and methionine are two sulfur-containing amino acids found in natural proteins and are essential amino acids for shrimp [26,27]. Nucleocytoplasmic transport pathway transports macromolecules to the correct organelles in an efficient and organized manner, which is essential for the proper functioning of eukaryotic cells [28]. Apoptosis is also an essential part of cellular development [29]. Amino acids are also important nutrients for the development of the embryo stored in the oocyte [30]. The above results suggested that *Lvit*-GIH2 might have an inhibitory effect on ovarian development.

On the other hand, the up-regulated DEGs mainly enriched in Folate biosynthesis, Glycerolipid metabolism, and Sphingolipid metabolism, which were associated with ovarian development. Folate accumulation is essential for methylation of DNA and histones during mammalian oocyte growth [31]. It is essential to the normal growth of juvenile Chinese mitten crabs, *Eriocheir sinensis* [32]. Lipids are preferable energy source for vitellogenesis during the ovarian maturation of crustaceans [33]. Lipid mobilization provides energy to the organism. It could be further converted to steroids, promoting Vg formation [34,35]. In the present study, the up-regulated DEGs mainly enriched in the upstream signaling factors of these signaling pathways. It could be speculated that the upstream factors of the signaling pathways that promote ovarian development increased to maintain the physiological progress. This result might be explained by compensating effect to maintain the normal ovarian development, which is disrupted by rGIH2.

Furthermore, we selected several DEGs for qRT-PCR validation to explore the effect of *Lvit-*GIH2 on ovarian development. It has been known that steroid levels change with the ovarian development [36], and estradiol 17-β (E2) is an important sex steroid hormone that promotes ovarian development in crustaceans [37–39]. Two isoforms of 17b-hydroxysteroid dehydrogenase 8 (17bHSD8) are associated with vertebrate steroidogenic pathways [40]. 17b-HSDs catalyze the last steps of steroid synthesis and its primary metabolism [41]. In the present study, we found that the expression of *17bHSD8* was significantly down-regulated by the addition of rGIH2, which revealed that *Lvit*-GIH2 might inhibit the ovarian development via suppressing E2 synthesis. In addition, as the precursor of prostaglandins (PG), arachidonic acid (AA) can stimulate ovarian maturation and oocyte differentiation to affect the growth and reproduction of female shrimp [42–44]. Cyclooxygenase (COX) catalyzes the production of PGs from AA and promotes ovarian development [45,46]. Lowering *COX* expression level suggested that *Lvit-*GIH2 might negatively regulate ovarian development.

Besides, we noted that GIH2 might be also involved in ovarian development via regulating insulin signal pathways. Insulin plays a crucial role in regulating growth, metabolism and reproduction in vertebrates [47]. Insulin-like hormones are widely distributed in invertebrates [48]. Recently, insulin-like androgenic hormone (IAG) was identified in *L*. *vittata* and it was found to function in promoting ovarian development [49]. In the mud crab *Scylla paramamosain*, an insulin-like receptors (IR) was founded to promote spermatogonial development [50]. The process of oocyte formation, growth and development is fundamental to ovarian development [51]. Insulin growth factor receptor (IGFR) plays a role in mediating insulin-like growth factor (IGF), which is important in regulating cell proliferation and normal development of the organism [52]. In comparison to the control group, the experimental group exhibited a trend towards decreased expression of *IGFR*, indicating that *Lvit-*GIH2 may exert an inhibitory role in ovarian development through the inhibition of *IGFR*.

Also, *Lvit*-GIH2 might suppress ovarian development by affecting energy supply. *CHH* has been shown to be a hormone in promoting gonadal development [25]. *CHH* in ovary was significantly down-regulated by the administration of rGIH2, which was rarely found in previous studies. Glycogen participates in macromolecular synthesis as a metabolic reserve in animals. Glycogen phosphorylase (GP) is a key enzyme involved in intracellular glycogen catabolism and catalyzes the first step in glycogen degradation [53]. Glycogen phosphorylase-like (*GPL*) showed a down-regulated expression pattern in the experimental group in this study, it was hypothesized that *Lvit-*GIH2 had an inhibitory effect on *GPL*. In summary, *Lvit-*GIH2 might exhibit the inhibitory effects on ovarian development in *L*. *vittata*.

## 5. Conclusion

The present study revealed that *Lvit-*GIH2 negatively regulated ovarian development in *L*. *vittata* based on the results of *in vitro* experiments and transcriptome sequencing. Further research is needed to elucidate the intricate mechanisms of *Lvit-*GIH2 on ovarian development and its broader biological functions.

## Supporting information

S1 Fig Multiple sequence alignment of putative mature peptides of CHH type II peptides. The six cysteine residues are shown in red background, the three putative disulfide bridges are connected with lines and the amidation sites are shown in blue background.
(TIF)

S2 Fig Phylogenetic tree of CHH type II peptides. The evolutionary history is inferred using the Neighbor-Joining method [54]. The bootstrap consensus tree inferred from 1000 replicates is taken to represent the evolutionary history of the taxa analyzed [55]. Branches corresponding to partitions reproduced in less than 50% bootstrap replicates are collapsed. The evolutionary distances are computed using the Poisson correction method and are in the units of the number of amino acid substitutions per site. The analysis involves 13 amino acid sequences. All positions containing gaps and missing data are eliminated. There are a total of 73 positions in the final dataset. Evolutionary analyses are conducted in MEGA6 [56]. *Lvit-GIH2* is marked with the red asterisks.
(TIF)

S3 Fig Prokaryotic expression and identification of rGIH2. (A) SDS-PAGE analysis of r*Lvit*-GIH2. The samples are run on 12% acrylamide gels. M: Marker. Lane 1, bacterial lysates of pET-His-transduced cells. Lane 2, bacterial lysates of pET-His-GIH2-transduced cells without IPTG induction. Lane 3, bacterial lysates of pET-His-GIH2-transduced cells with IPTG induction. Lane 4, bacterial lysates supernatant of pET-His-GIH2-transduced cells with IPTG induction. Lane 5, bacterial lysates precipitation of pET-His-GIH2-transduced cells with IPTG induction. (B) Western analysis with anti-His antibody of rGIH2 proteins. M: Marker. Lane 1: rGIH2. The arrowhead indicates the position of rGIH2. Positions of molecular weight markers (75, 25, 17, 11 kDa) are marked.
(TIF)

S4 Fig Analysis of DEGs in ovarian regions. (A) The heatmap of DEGs. (B) Volcano diagram of DEGs. The x-axis indicates the fold change, and the y-axis indicates the statistical significance of the differences. (C) Enriched Level 2 GO terms for DEGs. The x-axis indicates the Level 2 GO terms, and the y-axis indicates the number of DEGs.
(TIF)
