# Supplementary figures and images for: A new type II CHH neuropeptide involves ovarian development in the peppermint shrimp, Lysmata vittata
Source: PLoS One. 2024 Aug 1;19(8):e0305127. doi: 10.1371/journal.pone.0305127 (PMC11293640; doi:10.1371/journal.pone.0305127)

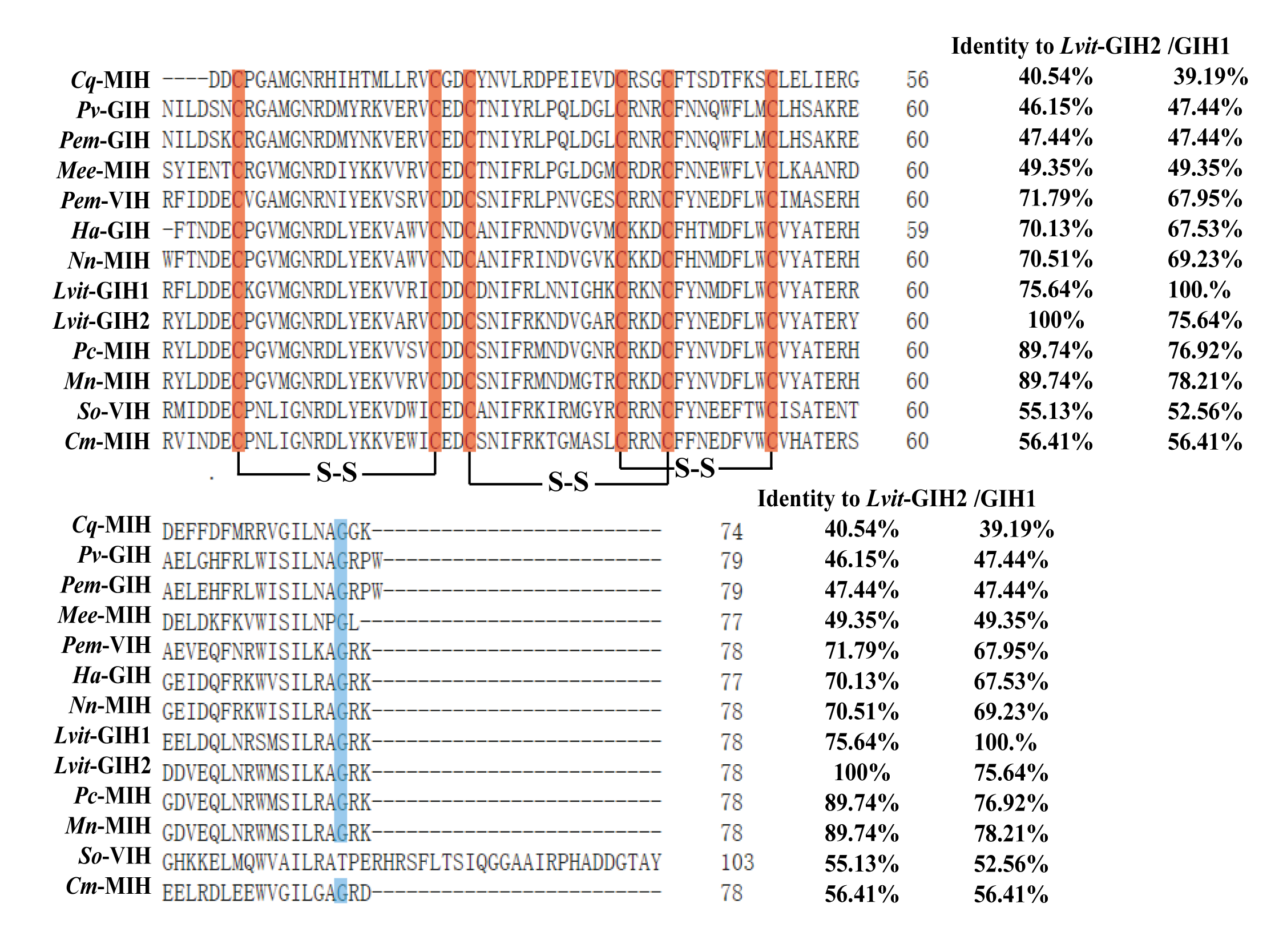

Supplement: S1 Fig — The six cysteine residues are shown in red background, the three putative disulfide bridges are connected with lines and the amidation sites are shown in blue background. (TIF) [file pone.0305127.s001.tif]

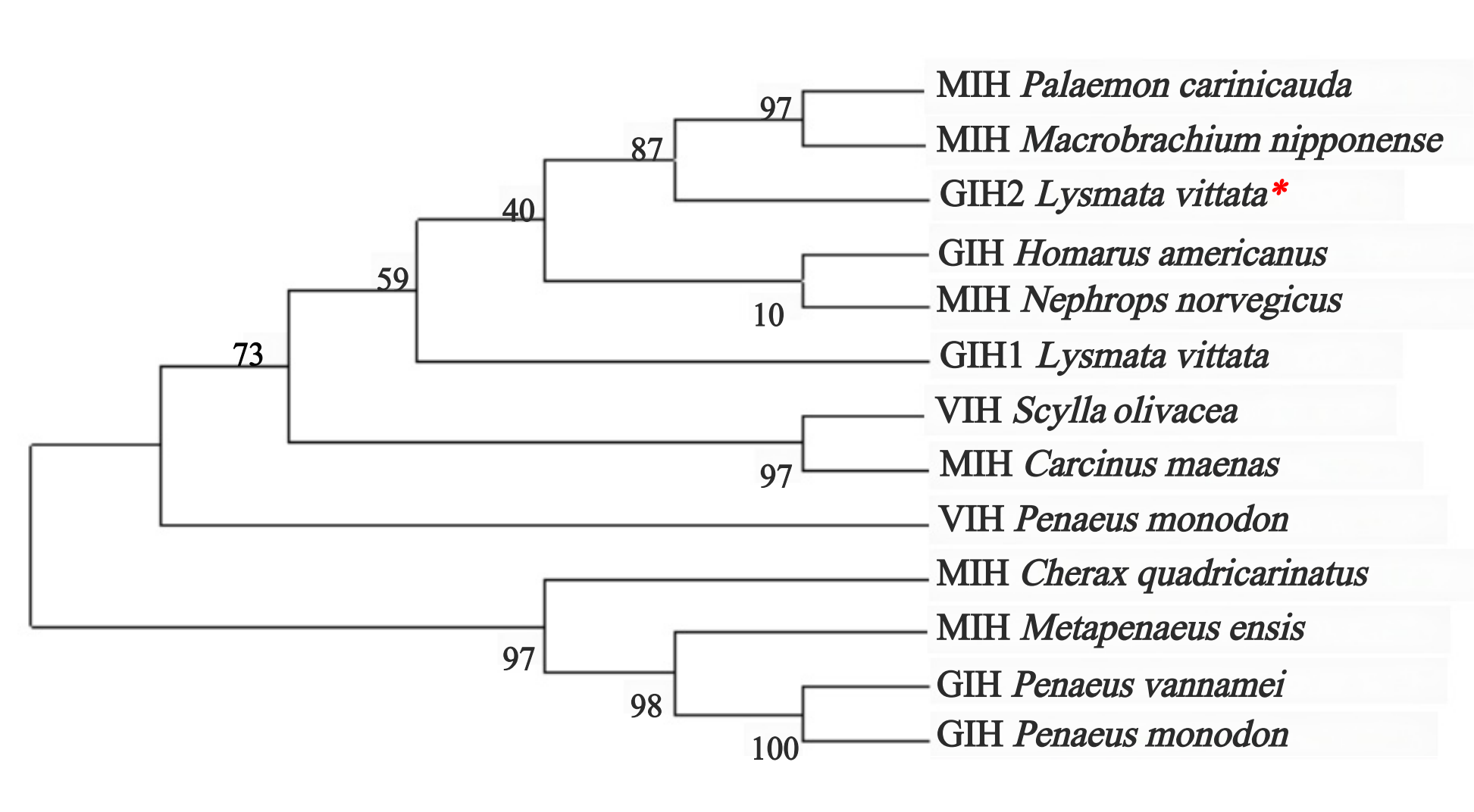

Supplement: S2 Fig — The evolutionary history is inferred using the Neighbor-Joining method [54]. The bootstrap consensus tree inferred from 1000 replicates is taken to represent the evolutionary history of the taxa analyzed [55]. Branches corresponding to partitions reproduced in less than 50% bootstrap replicates are collapsed. The evolutionary distances are computed using the Poisson correction method and are in the units of the number of amino acid substitutions per site. The analysis involves 13 amino acid sequences. All positions containing gaps and missing data are eliminated. There are a total of 73 positions in the final dataset. Evolutionary analyses are conducted in MEGA6 [56]. Lvit-GIH2 is marked with the red asterisks. (TIF) [file pone.0305127.s002.tif]

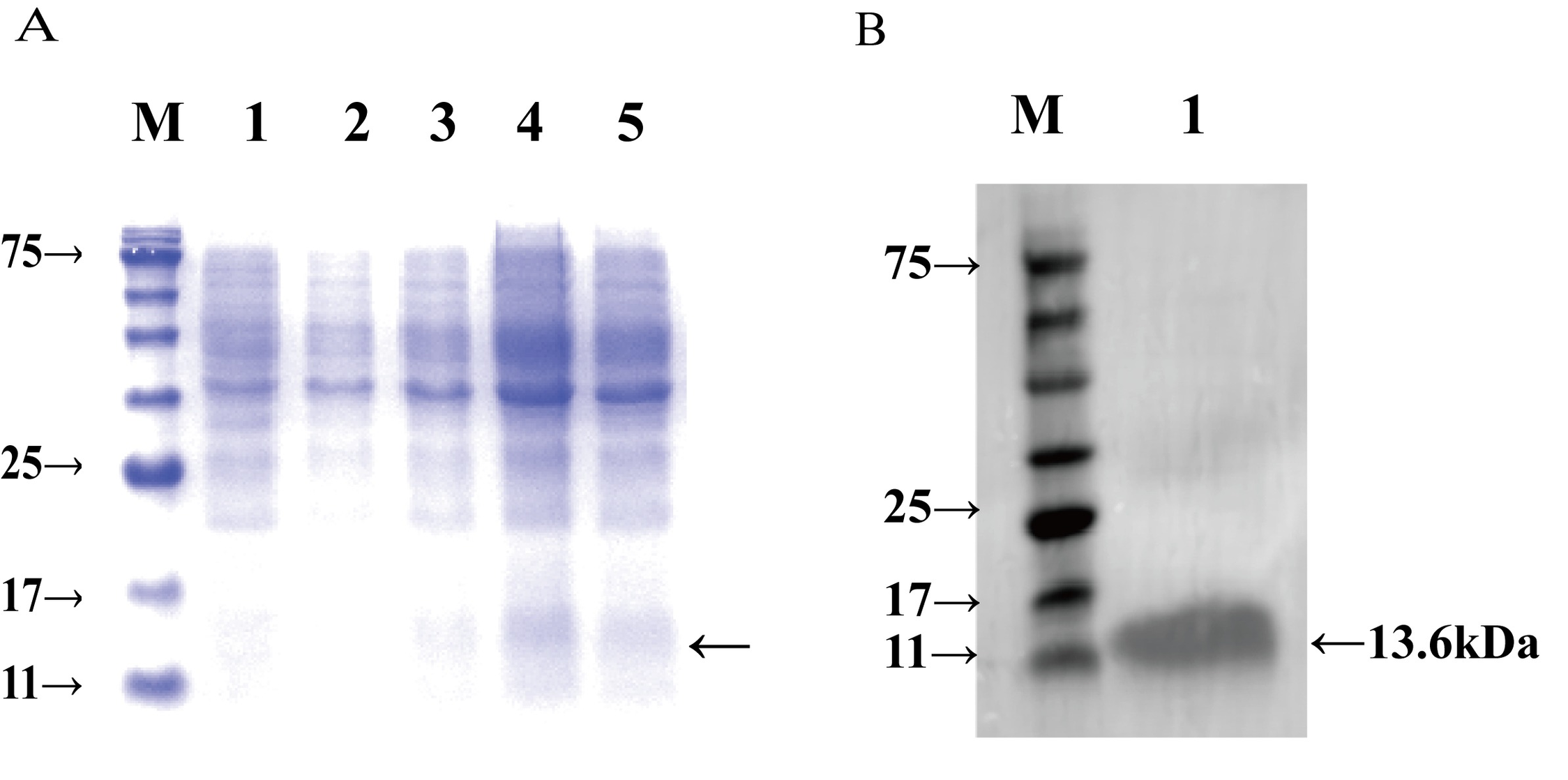

Supplement: S3 Fig — (A) SDS-PAGE analysis of rLvit-GIH2. The samples are run on 12% acrylamide gels. M: Marker. Lane 1, bacterial lysates of pET-His-transduced cells. Lane 2, bacterial lysates of pET-His-GIH2-transduced cells without IPTG induction. Lane 3, bacterial lysates of pET-His-GIH2-transduced cells with IPTG induction. Lane 4, bacterial lysates supernatant of pET-His-GIH2-transduced cells with IPTG induction. Lane 5, bacterial lysates precipitation of pET-His-GIH2-transduced cells with IPTG induction. (B) Western analysis with anti-His antibody of rGIH2 proteins. M: Marker. Lane 1: rGIH2. The arrowhead indicates the position of rGIH2. Positions of molecular weight markers (75, 25, 17, 11 kDa) are marked. (TIF) [file pone.0305127.s003.tif]

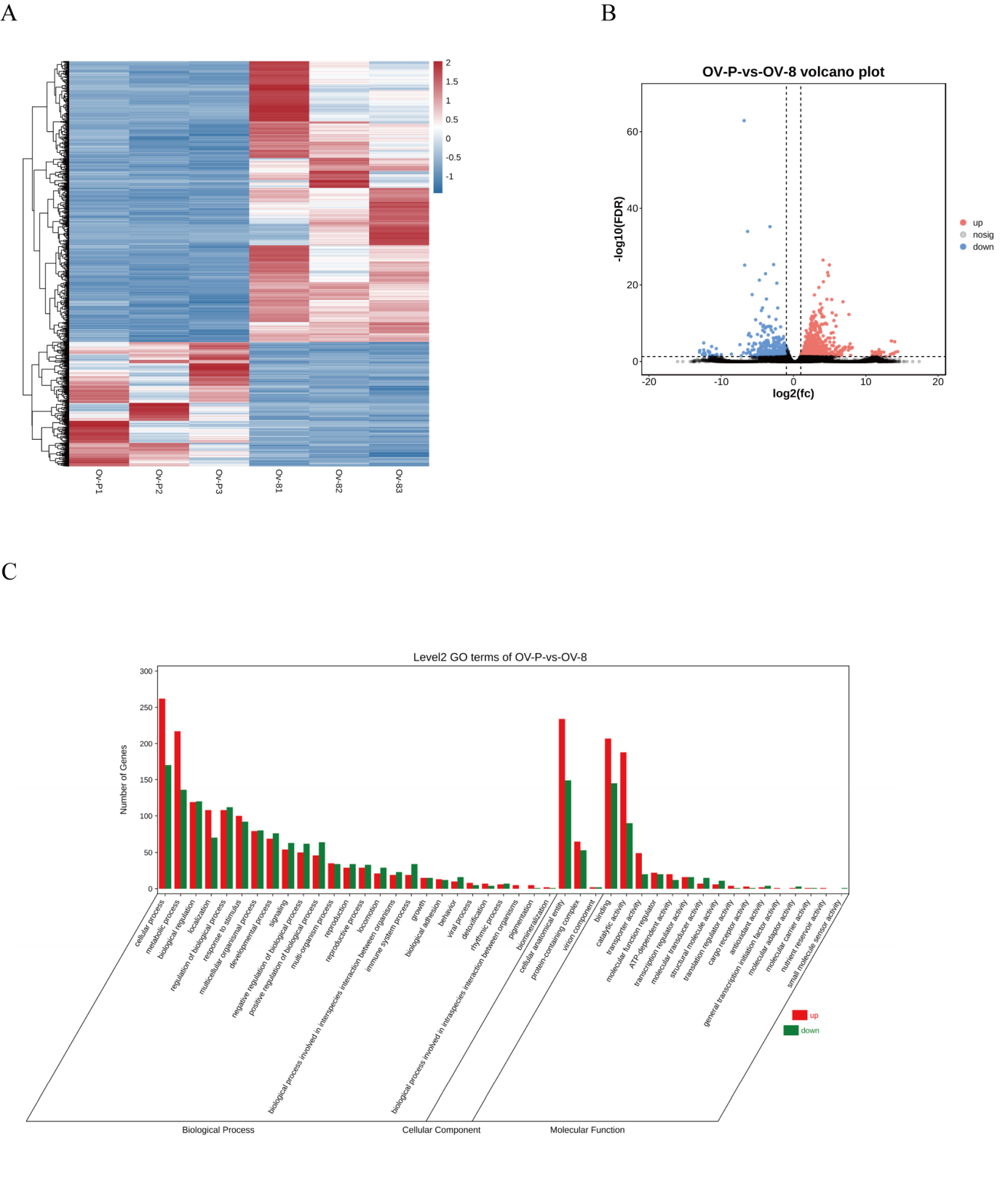

Supplement: S4 Fig — (A) The heatmap of DEGs. (B) Volcano diagram of DEGs. The x-axis indicates the fold change, and the y-axis indicates the statistical significance of the differences. (C) Enriched Level 2 GO terms for DEGs. The x-axis indicates the Level 2 GO terms, and the y-axis indicates the number of DEGs. (TIF) [file pone.0305127.s004.tif]
